# Coarse particulate matter air pollution in Porto Metropolitan area, Portugal: time series analysis, trends and implications for public policies

**DOI:** 10.1007/s11356-026-37546-w

**Published:** 2026-02-27

**Authors:** Simone Georges El Khouri Miraglia, Ronan Adler Tavella, Daniela Debone, Rui Moreira Barbosa, Flavio Manoel Rodrigues da Silva Júnior, Clara Lopes Ribeiro, Simone Morais, Marisa Alexandra Marques Freitas

**Affiliations:** 1 Institute of Environmental, Chemical and Pharmaceutical Sciences, Federal University of São Paulo, Diadema, Brazil; 2 ARIES, Antimicrobial Resistance Institute of São Paulo, São Paulo, Brazil; 3 CIIMAR/CIIMAR LA - Interdisciplinary Centre of Marine and Environmental Research, University of Porto, Matosinhos, Portugal Terminal de Cruzeiros do Porto de Leixões, Avenida General Norton de Matos, 4450-208; 4 Institute of Biological and Health Sciences, Federal University of Alagoas, Maceió, Brazil; 5 Institute of Environment and Development, University of Aveiro, Aveiro, Portugal; 6 ESS, Polytechnic Institute of Porto (ESS|P.PORTO), Porto, Portugal Rua Dr. António Bernardino de Almeida 400, 4200-072; 7 REQUIMTE/LAQV, ISEP, Polytechnic Institute of Porto, Porto, Portugal

**Keywords:** Air quality, Environmental health, PM_10_, Portugal, Spatial exposure, Urban health

## Abstract

Air pollution remains a persistent environmental and public health concern in urban regions. This study presents a 22-year analysis (2001–2022) of coarse particulate matter (PM_10_) concentrations across 15 monitoring stations in the Porto Metropolitan Area (PMA), Portugal. The stations, pre-classified by national authorities as traffic, background-urban, background-suburban, or industrial, were used to assess long-term temporal trends and spatial variability. Annual mean PM_10_ concentrations were calculated and linear regression was applied to evaluate trends. Differences between station types were tested using statistical analysis. The results revealed consistent decline in PM_10_ levels from the early 2000s to the mid-2010s, followed by stagnation or increases at several stations in the final years of the series. While higher concentrations were historically observed at traffic and industrial sites, no statistically significant differences in annual mean PM_10_ concentrations were found between stations of different classifications or within classifications, even when comparing distinct time periods. This homogeneity suggests widespread regional exposure, likely driven by shared emission sources, atmospheric transport, and urban development patterns across the PMA. When compared to World Health Organization (WHO) air quality guidelines, exceedances were frequent and persistent. From 2001 to 2011, nearly all stations with valid data exceeded the 2005 guideline of 20 µg/m^3^. Between 2012 and 2020, the exceedances gradually decreased, although several stations still surpassed the guideline. However, under the 2021 WHO guideline (15 µg/m^3^), all stations with valid data for 2021 and 2022 again exceeded the guideline. These findings highlight the need for integrated, long-term air quality policies.

**Clinical trial number:** Not applicable.

**Supplementary Information:**

The online version contains supplementary material available at 10.1007/s11356-026-37546-w.

## Introduction

Air pollution is the leading environmental risk factors to global human health, responsible for an estimated 8.1 million premature deaths per year, primarily due to cardiovascular and respiratory diseases (Health Effects Institute (HEI) 2024). Among air pollutants, coarse particulate matter (PM_10_), particles with an aerodynamic diameter ≤ 10 µm, has been widely associated with adverse health outcomes (Adar et al. 2014; Kim et al. 2015; Lu et al. 2015; Wu et al. 2018; Lei et al. 2023; Mohammadi et al. 2024; Miao et al. 2025; Tavella et al. 2025). Due to their deposition predominantly in the upper airways, PM_10_ particles can penetrate the upper respiratory tract and contribute to inflammatory responses, being associated with the exacerbating of conditions such as asthma, chronic obstructive pulmonary disease (COPD) and ischemic heart disease (IHD), although the magnitude and consistency of these associations are generally more pronounced for finer particulate fractions (Brunekreef and Forsberg 2005; Adar et al. 2014; Keet et al. 2018; Liu et al. 2020; Kaur et al. 2022; Guo et al. 2023). Particularly vulnerable populations include children, the elderly and individuals with preexisting conditions (Liu et al. 2018; Chen et al. 2019; Ziou et al. 2022).

Despite significant advances in emissions reduction across Europe following the implementation of mitigation measures, ambient air pollution remains a persistent public health challenge. According to the European Environment Agency (European Environment Agency (EEA) 2025), in 2022, exposure to air pollutants above WHO-recommended thresholds contributed to over 350,000 premature deaths across the European Union. Under the current EU Air Quality Directive (2008/50/EC), the annual limit value for PM_10_ is set at 40 µg/m^3^, with a daily limit of 50 µg/m^3^ not to be exceeded on more than 35 days per year. These standards have been transposed into Portuguese legislation through national air quality regulations and are formally met at the national level. However, Portugal follows the broader European pattern, particularly in urban and industrialized areas, where pollutant concentrations frequently approach or exceed health-based recommendations (Duarte et al. 2024; Pires 2025). Importantly, the EU limit values remain substantially less stringent than the updated WHO Air Quality Guidelines (2021), which recommend an annual PM_10_ concentration of 15 µg/m^3^ (World Health Organization (WHO) 2021). This limit is consistently surpassed in major Portuguese cities (Mendes et al. 2022; Duarte et al. 2024; Pires 2025; Miraglia et al. 2025), highlighting that regulatory guidance does not necessarily imply public health protection.

The health burden associated with particulate pollution in Portugal remains substantial. Studies using the AirQ + model estimate that long-term exposure to PM_2.5_ and NO_2_ contributes to over 5000 premature deaths per year, with mortality disproportionately concentrated in urban coastal regions such as Lisbon Metropolitan Area (LMA) and Porto Metropolitan Area (PMA) (Brito et al. 2022; Simões et al. 2025; Duarte et al. 2025). Recent comparative analyses show that the PMA exhibits a higher average cardiorespiratory mortality rate (202.94 deaths per 100,000) than the LMA (169.70 deaths per 100,000), with elevated PM_10_ concentrations being strongly associated with this excess mortality (Duarte et al. 2025). These findings underscore the need for targeted and evidence-based air quality management, particularly in metropolitan areas.

The PMA represents a complex urban landscape comprising 17 municipalities in northern Portugal. It includes highly urbanized cores, such as the city of Porto, where population density exceeds 5000 inhabitants/km^2^, as well as suburban, rural and forested peripheries. This urban–rural gradient is accompanied by a heterogeneous mix of pollution sources, including road traffic, industrial activities, biomass burning for residential heating and seasonal wildfires (Pires 2025; Simões et al. 2025). The latter, increasingly frequent and intense due to climate change, contribute episodically to significant PM₁₀ increases across the region (Pires 2025; Miraglia et al. 2025). The region’s topography and meteorology further complicate air quality dynamics. Coastal winds and summer sea breezes can transport pollutants across municipal boundaries, broadening population exposure even in areas distant from primary sources. As a result, wildfire smoke, industrial plumes and other airborne pollutants may affect urban centers not directly associated with their emission (Mendes et al. 2022; Duarte et al. 2025).

Given this complexity, investigating long-term trends in PM_10_ concentrations across the PMA is essential to understand how emission patterns and mitigation efforts have evolved over time. Moreover, analyzing differences by station classification, categorized according to predominant exposure settings in urban, suburban, traffic and industrial, can help identify whether specific exposure contexts are consistently associated with higher pollutant concentrations, and whether these differences are statistically significant. Such information is vital for targeting interventions in the areas where populations are most exposed and most at risk.

Long-term analyses are particularly relevant in this context, as they allow the evaluation of how regulatory shifts, technological advances and urban transformation processes have influenced air quality over time. Over the past two decades, increasingly stringent air quality standards, such as the WHO’s 2005 and 2021 guideline updates, have raised the bar for public health protection (World Health Organization (WHO) 2005; World Health Organization (WHO) 2021), compelling governments to adopt more effective emission control strategies. Simultaneously, structural and behavioral changes, ranging from transportation and industrial reforms to land use dynamics, urban expansion and residential heating practices, can significantly reshape pollution patterns (Landrigan 2017). Understanding how these factors have interacted to affect PM_10_ concentrations requires a temporal perspective that captures both gradual improvements and recent reversals. Therefore, investigating long-term patterns is not only important for tracking progress, but also for identifying persistent challenges and informing adaptive, forward-looking public policies.

In this context, this study conducts a 22-year time-series analysis of PM_10_ concentrations across 15 monitoring stations in the Porto Metropolitan Area. Understanding PM_10_ concentration patterns over time and between different urban contexts is essential to guide effective public policies for air quality and public health protection. Accordingly, the main objectives of this study are: (i) identify long-term trends and spatial patterns of PM_10_ considering 15 monitoring stations in the PMA, (ii) assess statistical differences in PM_10_ concentrations by station category, and (iii) explore implications for regional air quality management. By integrating long-term environmental monitoring data with statistical analysis, this study offers informative results on the effectiveness of past measures and the remaining challenges. These results are intended to support the design of targeted, evidence-informed public policies to improve air quality and health outcomes in the PMA and similar urban regions.

## Material and methods

### Study area

The study was conducted in the PMA, located in northwest Portugal along the Atlantic coast. The PMA comprises 17 municipalities, including the city of Porto and its surrounding urban, suburban and rural areas. It is the second-largest metropolitan region in the country, with a total area of 2040.31 km^2^ and a population of 1,743,272 inhabitants, according to the most recent national census (Instituto Nacional de Estatística (INE) 2021).

The region presents a marked urban–rural gradient in land use and population density. The city of Porto, the central municipality of the metropolitan area, has approximately 248,000 inhabitants in 41 km^2^ and a population density exceeding 6000 inhabitants/km^2^, forming a dense urban agglomeration with neighboring municipalities (Instituto Nacional de Estatística (INE) 2021). Other highly urbanized municipalities, such as Matosinhos and São João da Madeira, also exhibit population densities above 2900 inhabitants/km^2^, reflecting environments with a large potentially exposed population. In contrast, peripheral areas include lower-density suburban towns and predominantly rural municipalities. Municipalities such as Vale de Cambra (approximately 144 inhabitants/km^2^) and Arouca (approximately 63 inhabitants/km^2^) are characterized by low levels of urbanization and a more dispersed population (Instituto Nacional de Estatística (INE) 2021). In the Portuguese and broader European context, population densities below roughly 200–300 inhabitants/km^2^ are generally associated with rural or low-urbanization settings, whereas densities above 1500 inhabitants/km^2^ indicate highly urbanized environments. These contrasts illustrate the pronounced spatial heterogeneity in population distribution across the PMA.

Climatically, the PMA is classified as Csb under the Köppen–Geiger system (Beck et al. 2023), indicating a Mediterranean climate with mild, wet winters and warm, dry summers. Meteorological conditions such as sea breezes, Atlantic westerlies, temperature inversions and occasional Saharan dust transport influence local air quality dynamics and the dispersion or accumulation of air pollutants across the region (Pires 2025). These factors, combined with the mix of emission sources across the PMA create a complex setting for ambient particulate matter levels.

### Air quality monitoring and data collection

To characterize PM_10_ levels across the PMA, data from 15 active air quality monitoring stations, operating between January 2001 and December 2022, were compiled. These stations are part of the national air quality monitoring network, managed by the Portuguese Environment Agency (Agência Portuguesa do Ambiente, APA), which ensures standardized procedures and data quality. The data were retrieved from the QualAr platform, the official national system for air quality information (QualAr 2024).

Although several pollutants may be monitored at these stations, including O_3_, NO_2_, CO, SO_2_, PM_10_, and PM_2.5_, the availability of multi-pollutant data is heterogeneous across stations and over time. PM_10_ was selected as the focus of this study because it is the only pollutant monitored at all 15 stations at some point during the study period, allowing a consistent long-term and spatially complete regional analysis. In contrast, monitoring of other criteria pollutants was more limited in terms of spatial coverage and temporal continuity, particularly for PM_2.5_, which was measured at only two stations during restricted time intervals.

**Fig. 1:**
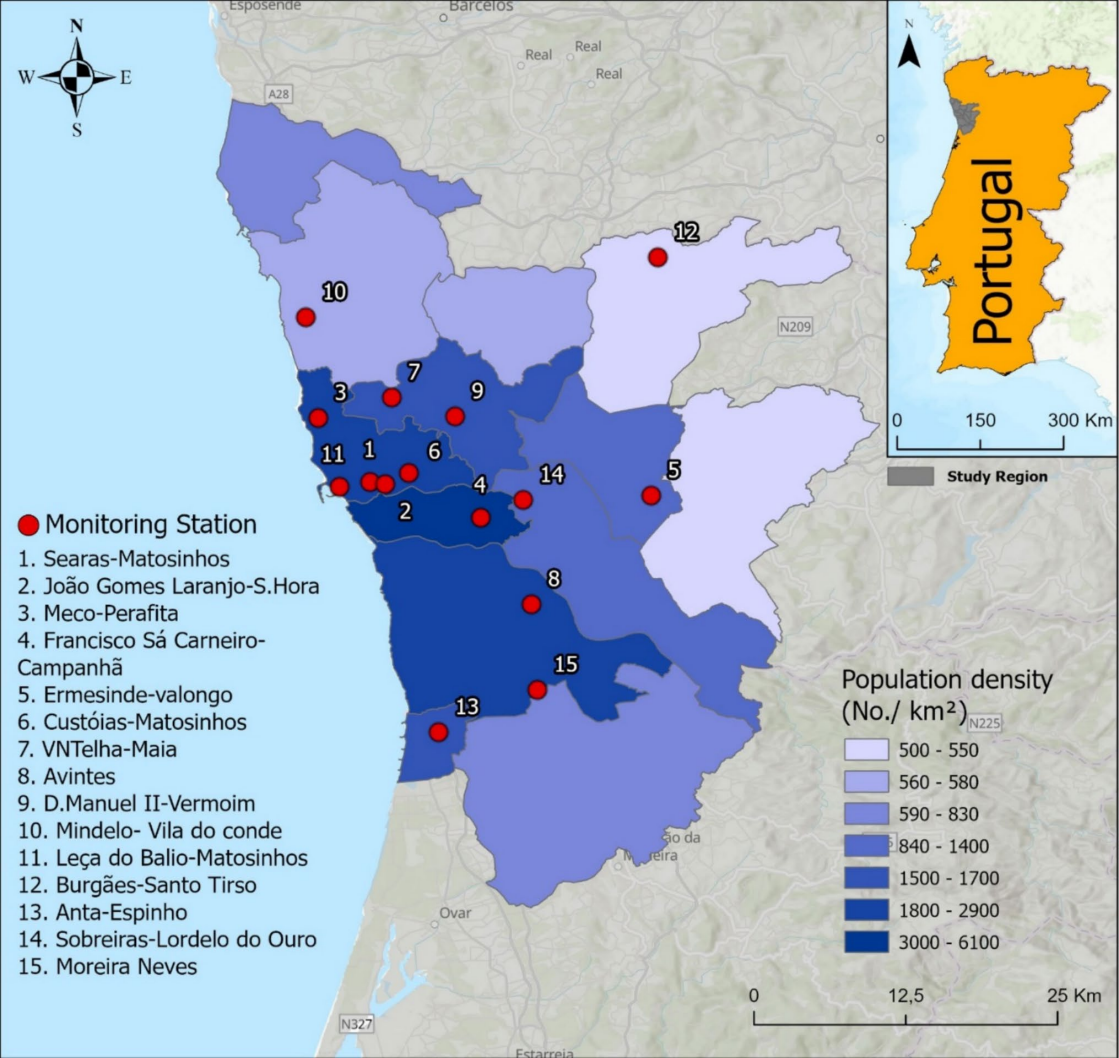
Geospatial distribution of the 15 air quality monitoring stations (red dots) in the PMA, shown in relation to population density (inhabitants per km^2^). Different shades of blue represent population density ranges

The 15 stations considered in this study correspond to all air quality monitoring stations operating within the PMA during the study period, and their geographic distribution reflects the region’s urban–rural gradient, ensuring representative spatial coverage across municipalities. Figure 1 shows the geospatial distribution of the monitoring stations overlaid on the population density (inhabitants/km^2^) of the PMA.

The stations are classified according to predominant exposure settings into four categories: (i) traffic stations, which are located near major roads and primarily capture emissions from vehicular activity; (ii) urban background stations, situated in densely populated areas but away from direct emission sources, representing general urban exposure; (iii) suburban background stations, positioned in intermediate-density zones between urban centers and rural areas; and (iv) industrial stations, located in proximity to large industrial complexes or areas with significant stationary sources of pollution. The classification of each air quality station is presented in Table 1.

**Table 1:** Air quality monitoring station classification according to the QualAr system (QualAr 2024), operational period, and mean values ± standard deviation of PM_10_ calculated over the active monitoring period of each air quality station in the PMA

Air quality station	Classification	Number in Fig. 1	Active period	PM_10_ (µg/m^3^)
Custóias – Matosinhos	Background—Suburban	6	2001–2022	28.63 ± 11.23
VNTelha – Maia	Background—Suburban	7	2001–2022	27.69 ± 10.83
Leça do Balio – Matosinhos	Background—Suburban	11	2001–2022	28.42 ± 11.30
Mindelo – Vila do conde	Background—Suburban	10	2010–2022	19.18 ± 4.95
Anta – Espinho	Background—Suburban	13	2011–2022	21.40 ± 8.81
Ermesinde – Valongo	Background—Urban	5	2001–2022	30.51 ± 9.12
Sobreiras – Lordelo do Ouro	Background—Urban	14	2009–2022	23.37 ± 4.48
Burgães – Santo Tirso	Background—Urban	12	2009–2022	17.79 ± 5.12
Avintes	Background—Urban	8	2010–2022	19.33 ± 5.14
D. Manuel II – Vermoim	Traffic—Urban	9	2001–2022	29.61 ± 13.57
Franscico Sá Carneiro – Campanhã	Traffic—Urban	4	2001–2022	34.35 ± 23.66
João Gomes Laranjo – S. Hora	Traffic—Urban	2	2001–2022	32.00 ± 15.67
Moreira Neves – Castelões de Cepeda	Traffic—Urban	15	2004–2022	23.62 ± 13.16
Searas—Matosinhos	Industrial—Urban	1	2013–2022	19.92 ± 2.91
Meco – Perafita	Industrial—Suburban	3	2002–2022	31.13 ± 10.10

PM_10_ concentrations were recorded as 24-h average values (µg/m^3^) at each station. These daily values were used to calculate annual means for each station and year, enabling both temporal and spatial comparisons. Annual means were chosen as a primary metric because they smooth out short-term fluctuations and are commonly used for trend analysis and comparison to guideline values. In calculating annual means, completeness criteria consistent with regulatory guidelines were followed — requiring that over 70% of days in a year had valid data for the mean to be considered representative. In a few cases where a station had significant data gaps in a particular year (due to station downtime or other issues), that year-station was excluded from trend analysis to avoid bias.

### Statistical analysis

For the statistical analysis, annual mean PM_10_ concentration were used as the primary input for all subsequent evaluations. To evaluate temporal trends, an exploratory analysis of the annual mean values for each station was conducted, allowing visualization of general patterns, trends and potential outlier years. Linear trends were quantified using linear regression, from which the slope coefficients to estimate the rate of change in PM_10_ concentrations over time were extracted. Associated *p*-values were used to assess the statistical significance of these trends.

To compare PM_10_ concentrations across station classifications and assess whether differences were statistically significant, hypothesis testing procedures were applied. These comparisons were performed both (i) among stations of the same classification and (ii) across all station types. Prior to hypothesis testing, the annual mean PM10 data from the individual monitoring stations were assessed for normality and were found to be approximately normally distributed, supporting the use of parametric methods. When comparing groups with more than two stations, one-way analysis of variance (ANOVA) was used with Welch and Brown-Forsythe adaptations to account for unequal variances. In cases where only two stations were available within a classification, such as the industrial category, comparisons were performed using unpaired *t*-tests. These analyses were also applied to sub-periods (e.g., 2001–2009 vs. 2010–2022) to assess whether patterns and differences changed over time. The definition of sub-periods was based on structural changes in the monitoring network, particularly the implementation of new active stations. For instance, if a given classification had only two stations operating until 2009 and three additional stations began operating in 2010, the sub-periods were defined as 2001–2009 and 2010–2022.

All statistical analyses were conducted using Statistica software, version 12.0. Graphical representations of trends were produced using GraphPad Prism, version 8.0. For all statistical tests, a significance level of 0.05 was adopted.

## Results

**Fig. 2:**
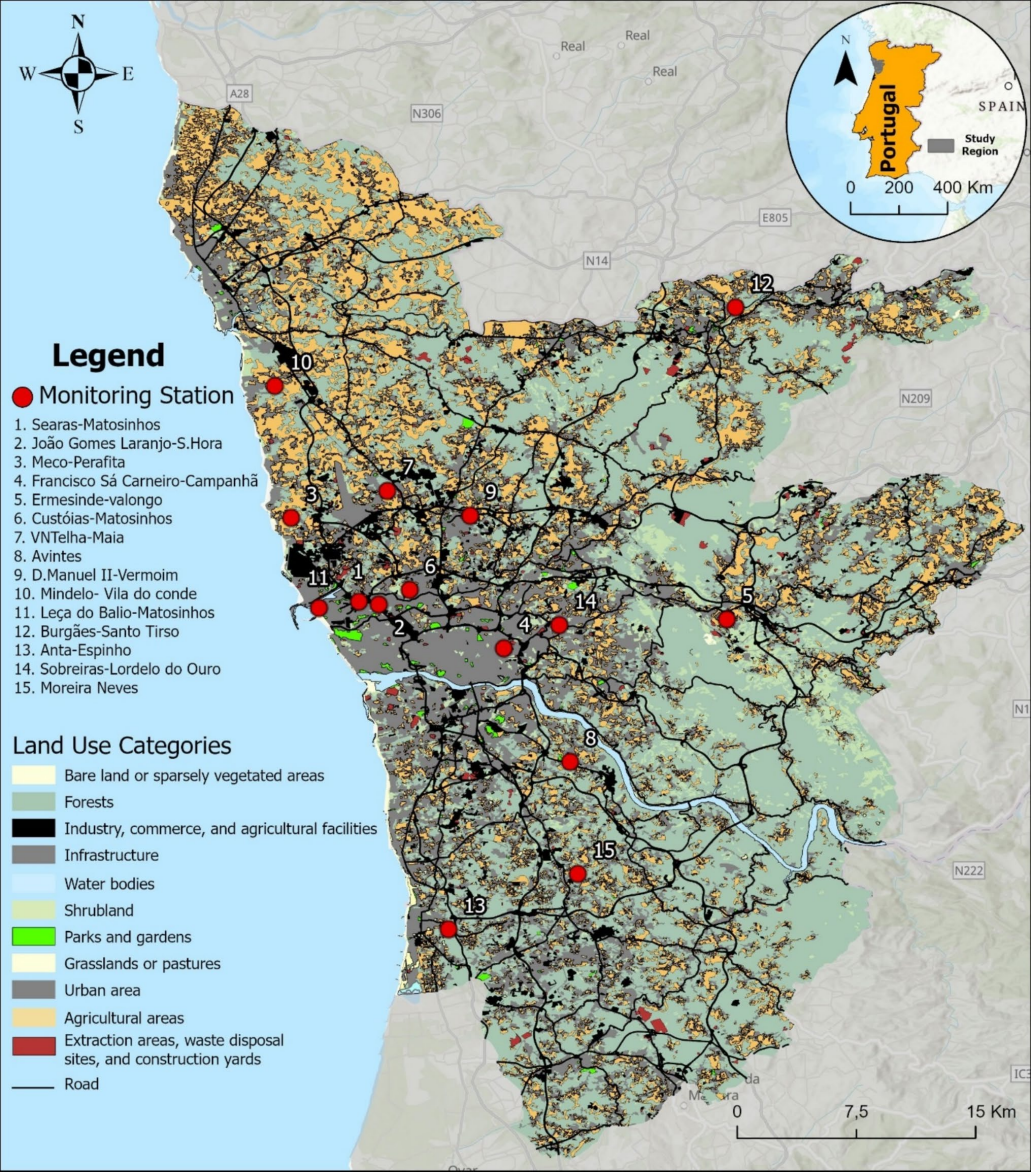
Spatial distribution of land use categories in the PMA and location of the air quality monitoring stations (red dots). Land use classification was based on the 2018 Land Use and Land Cover Map (COS 2018), according to the Directorate-General for Territory

Figure 2 presents the spatial distribution of land use categories across the PMA, along with the location of the air quality monitoring stations. The classification of land use includes urban areas, infrastructure, forests, agricultural zones, industrial and commercial facilities, among others. It is possible to observe that most air quality stations are located in or near dense urbanized areas and transportation corridors, consistent with population distribution and the location of major emission sources. Several suburban and background stations are situated near forested or mixed land-use regions, while industrial stations are clearly positioned within or adjacent to areas designated for industrial activity. This spatial arrangement reflects the diversity of environmental exposure contexts considered in the monitoring network.

A total of 15 air quality monitoring stations were evaluated, classified into four types: five background-suburban, four background-urban, four traffic and two industrial stations. The longest monitoring period, spanning the full 22 years (2001–2022), corresponded to seven stations: Custóias – Matosinhos, VNTelha – Maia, Leça do Balio – Matosinhos, Ermesinde – Valongo, D. Manuel II – Vermoim, Francisco Sá Carneiro – Campanhã, and João Gomes Laranjo – S. Hora. In contrast, the station with the shortest monitoring period was Searas – Matosinhos, active from 2013 to 2022. The highest total average PM_10_ concentration was recorded at the traffic station Francisco Sá Carneiro – Campanhã (34.35 ± 23.66 µg/m^3^), while the lowest was observed at the background-urban station in Burgães – Santo Tirso (17.79 ± 5.12 µg/m^3^), as summarized in Table 1.

**Fig. 3:**
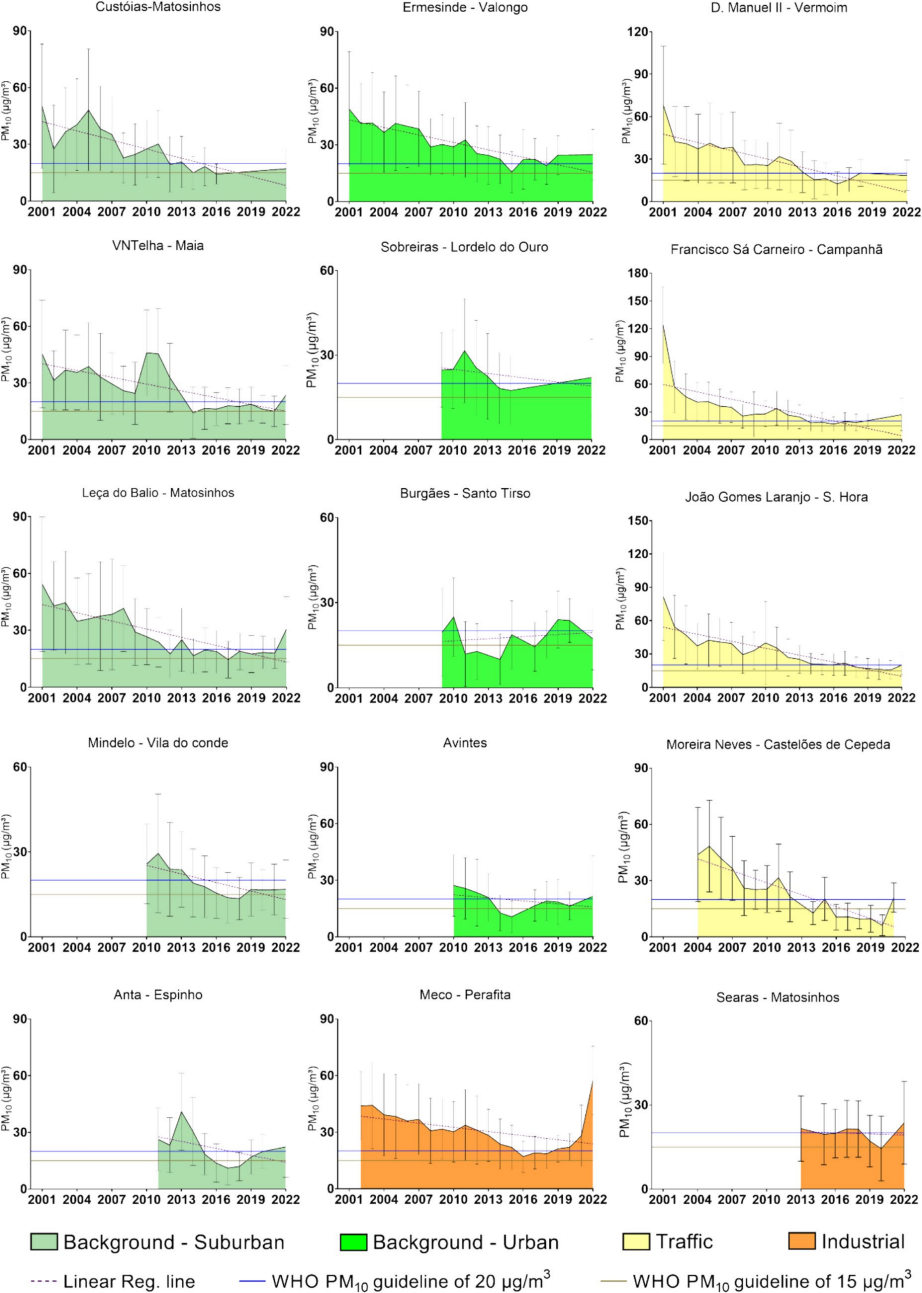
Annual mean PM₁₀ concentrations (µg/m^3^) with standard deviation for each of the 15 air quality monitoring stations, categorized by type: background-suburban (green), background-urban (bright green), traffic (yellow) and industrial (orange). The data cover the operational period of each station between 2001 and 2022. The dotted line represents the linear trend based on available annual means for each station. Horizontal lines indicate the WHO annual guideline values for PM_10_: 20 µg/m^3^ (blue line) and 15 µg/m^3^ (brown line)

Table S1 (Supplementary Material) presents the annual mean PM_10_ concentrations (µg/m^3^) recorded at each of the 15 air quality monitoring stations in the PMA over their respective operational periods between 2001 and 2022. These values, which are visually synthesized in Fig. 3, reflect substantial temporal and spatial variability in PM_10_ levels across the region, influenced by local emission sources, land use, and meteorological conditions. Several stations that operated throughout the entire 22-year period consistently recorded some of the highest annual concentrations, particularly in the early 2000s. A general decline in PM_10_ concentrations over time is observable in many stations, particularly after 2010. For example, Custóias – Matosinhos decreased from values above 50 µg/m^3^ in the early 2000 s to levels consistently below 25 µg/m^3^ in the last decade. Similarly, Francisco Sá Carneiro – Campanhã, originally registering the highest value in the dataset (124.08 µg/m^3^ in 2001), showed a marked reduction over the years, despite continued variability. However, some fluctuations and episodic increases were observed in the later years at specific stations, such as Meco – Perafita, which reached its peak value (57.35 µg/m^3^) in 2022.

Among the background-suburban stations, average PM_10_ concentrations ranged from 50.21 µg/m^3^ to 9.97 µg/m^3^. For the background-urban stations, values ranged from 54.35 µg/m^3^ to 11.10 µg/m^3^. Traffic stations exhibited a broader range, from 124.08 µg/m^3^ to 6.20 µg/m^3^. Industrial stations showed values between 44.25 µg/m^3^ and 14.47 µg/m^3^. Overall, the highest PM_10_ concentrations were recorded during the early years of the time series, particularly at the following stations: Custóias – Matosinhos (background-suburban), Sobreiras – Lordelo do Ouro (background-urban), Francisco Sá Carneiro – Campanhã (traffic), and Meco – Perafita (industrial) (Fig. 3).

It is important to assess these trends in light of international health-based standards. Between 2001 and 2020, the WHO guideline for annual PM_10_ exposure was 20 µg/m^3^ (World Health Organization (WHO) 2005). From 2001 to 2011, all valid station measures exceeded this limit in every year, with only two exceptions: Burgães – Santo Tirso, which reported concentrations below 20 µg/m^3^ in 2009 and 2011. From 2012 onward, modest improvements were observed: in 2012, 11 of 14 valid stations exceeded the 20 µg/m^3^ guideline; in 2013, all valid stations exceeded it; in 2014, the number dropped to 4 of 14; in 2015, 3 of 15 stations were above the limit; in 2016, 2 of 12; in 2017, 3 of 13; in 2018, 2 of 13; in 2019, 4 of 12; and in 2020, only 2 of 10 exceeded the 20 µg/m^3^ limit, clearly demonstrating regional improvements in air quality. Under the stricter 2021 WHO guideline (15 µg/m^3^) (World Health Organization (WHO) 2021), compliance dropped substantially. In 2021, only 6 stations had valid data, and all exceeded the 15 µg/m^3^ limit, although VNTelha – Maia reported concentrations close to this threshold. In 2022, of the 14 stations with valid data, none met the WHO 2021 standard, confirming that PM_10_ concentrations across the PMA remained above levels considered health-protective under current international recommendations.

**Fig. 4:**
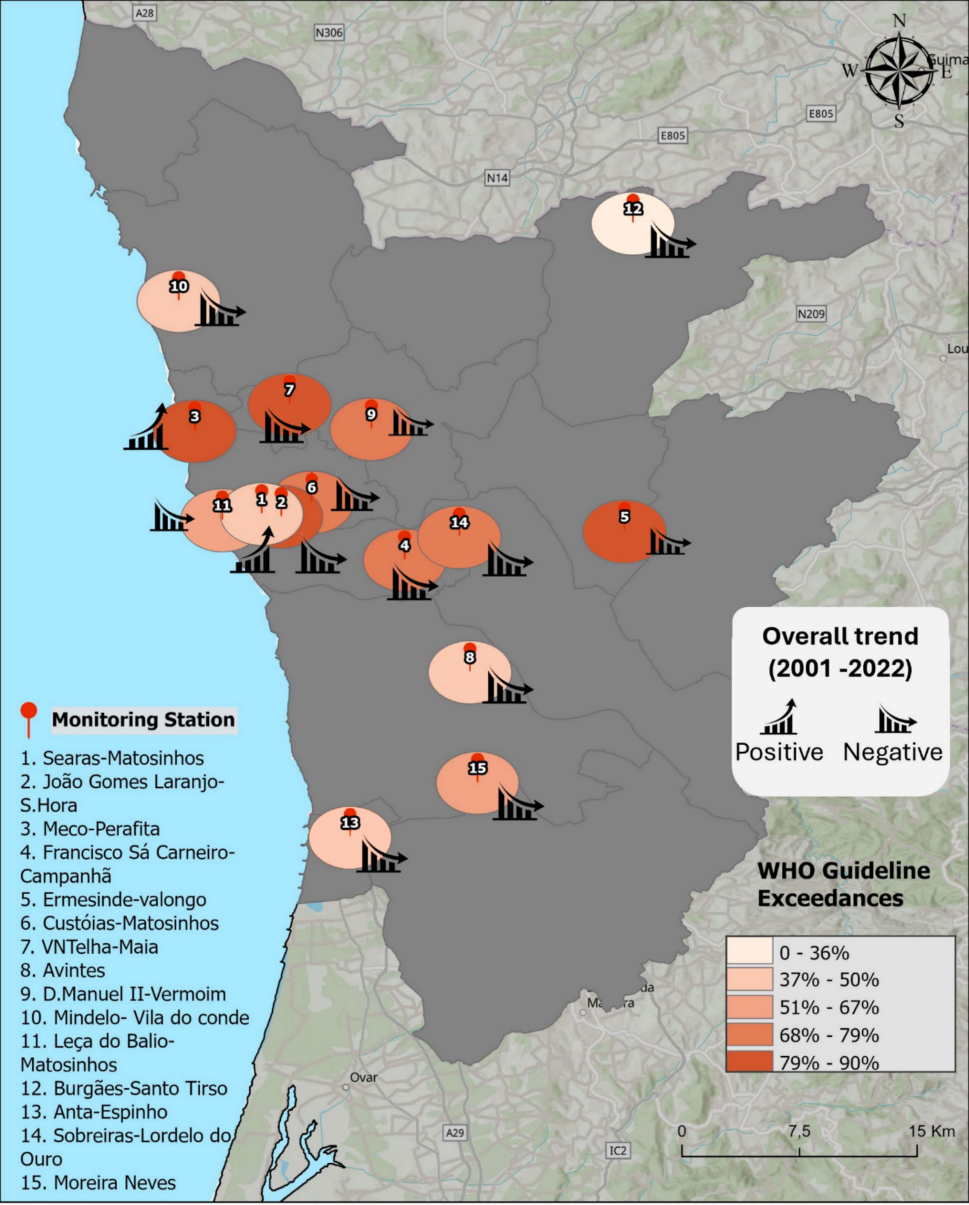
Spatial distribution of PM_10_ exceedances of WHO annual guideline values and overall direction of long-term trends (2001–2022) across the PMA. Circle color represents the percentage of years with valid data in which annual mean PM_10_ concentrations exceeded WHO guidelines at each monitoring station. Trend symbols indicate the direction of long-term trends (positive or negative)

An additional spatial perspective on guideline exceedances is provided by Fig. 4, which summarizes, for each monitoring station, the proportion of years exceeding WHO guideline values alongside the overall direction of long-term PM_10_ trends, highlighting spatial differences in the persistence of exceedances across the PMA. The proportion of years exceeding WHO guideline values ranged from approximately 36% to 90% across the monitoring stations, with consistently high exceedance frequencies observed across all station categories. Detailed station-specific values are reported in Table S2 (Supplementary Material). For most stations, WHO guideline exceedances occurred in nearly half or more of the years with valid data. Overall, the results underscore the heterogeneous behavior of PM_10_ concentrations across the PMA, shaped by the interaction between emission patterns, land use and meteorological dynamics. This variability highlights the need for localized approaches to air quality management and supports the use of classification-based station comparisons, as explored in subsequent analyses.

Figure 3 also presents the linear trends from 2001 to 2022 for the characterized 15 air quality monitoring stations in the PMA. Inter-annual variability is represented by standard deviation bars, and trend lines reflect the direction and magnitude of linear changes over time based on available data for each site. The corresponding linear regression coefficients, confidence intervals, and *p*-values are summarized in Table 2. Overall, most monitoring stations exhibited negative trends in annual mean PM_10_ concentrations over the study period Statistically significant decreasing trends (*p* < 0.05) were identified at 10 of the 15 stations, including all traffic stations, most background-suburban stations, one background-urban station (Ermesinde – Valongo), and one industrial station (Meco – Perafita). Five stations did not present statistically significant trends over their respective monitoring periods, namely Anta – Espinho, Sobreiras – Lordelo do Ouro, Burgães – Santo Tirso, Avintes, and Searas – Matosinhos. Notably, all stations without statistically significant trends-initiated PM_10_ monitoring from 2009 onwards, resulting in shorter and less continuous time series, which may have limited the ability to detect long-term linear trends. Importantly, none of the stations exhibited a statistically significant positive trend in annual mean PM_10_ concentrations during the evaluated period.

**Table 2:** Linear regression results for annual mean PM_10_ concentrations at the 15 air quality monitoring stations in the PMA. Statistically significant trends are defined as *p* < 0.05

Air quality station	Classification	Active period	Slope (µg/m^3^·year^−1^)	95% CI	*p* value
6—Custóias – Matosinhos	Background—Suburban	2001–2022	− 1.609	− 2.249 to − 0.969	< 0.001
7—VNTelha – Maia	Background—Suburban	2001–2022	− 1.201	− 1.730 to − 0.672	< 0.001
11—Leça do Balio – Matosinhos	Background—Suburban	2001–2022	− 1.445	− 1.897 to − 0.994	< 0.001
10—Mindelo – Vila do conde	Background—Suburban	2010–2022	− 1.005	− 1.521 to − 0.489	0.001
13—Anta – Espinho	Background—Suburban	2011–2022	− 1.230	− 2.901 to 0.441	0.130
5—Ermesinde – Valongo	Background—Urban	2001–2022	− 1.318	− 1.667 to − 0.969	< 0.001
14—Sobreiras – Lordelo do Ouro	Background—Urban	2009–2022	− 0.497	− 1.482 to 0.487	0.262
12—Burgães – Santo Tirso	Background—Urban	2009–2022	0.246	− 0.6211 to 1.113	0.537
8—Avintes	Background—Urban	2010–2022	− 0.540	− 1.437 to 0.357	0.207
9—D. Manuel II – Vermoim	Traffic- Urban	2001–2022	− 1.958	− 2.565 to − 1.352	< 0.001
4—Franscico Sá Carneiro – Campanhã	Traffic- Urban	2001–2022	− 2.637	− 4.046 to − 1.228	0.001
2—João Gomes Laranjo – S. Hora	Traffic- Urban	2001–2022	− 2.099	− 2.654 to − 1.544	< 0.001
15—Moreira Neves – Castelões de Cepeda	Traffic- Urban	2004–2022	− 2.125	− 2.731 to − 1.520	< 0.001
1—Searas—Matosinhos	Industrial- Urban	2013–2022	− 0.127	− 1.131 to 0.871	0.762
3—Meco – Perafita	Industrial- Suburban	2002–2022	− 0.733	− 1.431 to − 0.034	0.041

Complementing these results, Fig. 4 provides a spatial overview of the overall long-term PM_10_ trends across the PMA, highlighting marked differences in the magnitude and direction of change between monitoring stations. Although declining trends predominated across most of the metropolitan area, the relative magnitude of long-term change varied substantially among sites. Overall reductions ranged from pronounced decreases exceeding 70% at several traffic stations to more modest declines at some background-urban and suburban stations. A more detailed quantification of these changes is provided in Table S2 (Supplementary Material), which reports relative PM_10_ concentration changes for the overall study period as well as for the 2001–2011 and 2012–2022 sub-periods. These data indicate that, while most stations experienced substantial reductions over the full study period, the rate and consistency of decline were not uniform over time. Several stations showed steeper reductions during the earlier years of monitoring, followed by attenuated declines or partial rebounds in the most recent decade. These findings further highlight the heterogeneous temporal dynamics across individual stations and reinforce both an overall tendency toward declining PM_10_ levels across the PMA and the importance of contextual interpretation when assessing long-term air quality patterns.

Statistical analyses indicated no significant differences in average PM_10_ concentrations between stations of the same classification in any of the evaluated time frames, with all *p*-values exceeding the 0.05 threshold (Table 3). For background-suburban stations, no significant variation was observed for the 2001–2009 and 2010–2022 periods (*p* = 0.24 and *p* = 0.72, respectively). Similarly, background-urban stations from 2009 to 2022 showed no significant differences, although results approached significance (*p* = 0.07). Traffic stations also exhibited no significant differences in either the early (2001–2003) or extended (2004–2022) periods (*p* = 0.58 and *p* = 0.67, respectively). For industrial stations, evaluated via unpaired *t*-test, no significant differences were detected (*p* = 0.21). When all stations were considered together, no significant spatial variation was observed for the 2002–2009 and 2010–2022 periods (*p* = 0.32 and *p* = 0.31, respectively), as detailed in Table 3. These results suggest that, within each category and time frame, PM_10_ concentrations remained statistically similar across stations of the same type.

**Table 3:** Statistical analysis of PM_10_ concentration differences across stations by type (background-suburban, background-urban, traffic, and industrial)

Station type	Comparison period	N of years^a^	Statistical difference (*p* value < 0.05)
Background—Suburban	2001–2009	9	No (*p* = 0.24)
Background—Suburban	2010–2022	6	No (*p* = 0.72)
Background – Urban	2009–2022^b^	6	No (*p* = 0.07)
Traffic	2001–2003	3	No (*p* = 0.58)
Traffic	2004–2022	14	No (*p* = 0.67)
Industrial	2013–2022^b^	8	No (*p* = 0.21)
All air quality stations	2002–009	7	No (*p* = 0.32)
All air quality stations	2010–2022	7	No (*p* = 0.31)

^a^ Numbers of possible years used for the comparison

^b^ Prior to this period only one air quality station of this type was active, preventing possible comparisons between stations

## Discussion

This study provides a robust long-term evaluation of PM_10_ concentrations in the PMA, using 22 years of validated data from a spatially distributed network of air quality monitoring stations. The analysis revealed a general decline in annual mean PM_10_ levels at most stations, particularly from the early 2000 s through the mid-2010s. This downward trend likely reflects the cumulative effects of air quality and climate-related policies, including the implementation of international commitments such as the Kyoto Protocol (United Nations Framework Convention on Climate Change (UNFCCC) 1998), European regulatory frameworks such as Directive 2001/77/EC (European Parliament and Council of the European Union 2001), promoting electricity generation from renewable energy sources, and national policy instruments. In Portugal, these improvements have been further supported by the National Strategy for Energy and Climate, enacted through Council of Ministers Resolution No. 169/2005, of 24 October (Portugal 2005). The progressive deployment of electric mobility initiatives and regulatory measures, modernization of vehicle fleets, improvements in industrial emission controls and broader urban environmental interventions (Ribeiro and Mendes 2022; Coelho et al. 2023), has certainly contributed to reducing transport sector emissions and mitigating particulate matter pollution in the PMA.

Despite these long-term improvements, in recent years this decline has plateaued or even reversed at several sites, with some stations, such as Meco – Perafita and Sobreiras – Lordelo do Ouro, showing renewed increases in mean concentrations. These temporal fluctuations suggest that progress in air quality has not been linear and may be susceptible to episodic pollution events or evolving local emission patterns. In particular, the slightly positive slope observed at Burgães – Santo Tirso, as well as transient increases detected at multiple stations during 2019–2020, reflects the influence of a limited number of years with higher concentrations rather than a sustained upward trend. Inter-annual variability and occasional PM_10_ increases during this period are likely linked, at least in part, to regional wildfire activity. In Portugal, the 2019 fire season was marked by more than 10,000 wildfires and over 42,000 hectares burned, among the highest annual burned areas in recent years, with significant events affecting northern districts, including the PMA (San-Miguel-Ayanz et al. 2020; Instituto da Conservação da Natureza e das Florestas (ICNF) 2020). Intense wildfire seasons are associated with elevated particulate matter concentrations at regional scales (Fernandes et al. 2022; Miraglia et al. 2025), and while such episodic smoke events do not alter the overarching long-term decline in ambient PM_10_, they can elevate annual mean concentrations at specific stations, contributing to departures from linear trends. Importantly, no statistically significant differences in mean annual concentrations were observed between monitoring stations of the same classification (e.g., traffic, background-urban), or across all stations combined within the same period. This finding suggests that, despite differences in local land use and station classification, PM_10_ levels were relatively homogeneous across the PMA. This limited spatial differentiation may be attributed to shared regional sources, atmospheric transport mechanisms, and widespread exposure across urban environments (Pires 2025).

To further contextualize these results, when benchmarked against international air quality standards, the observed PM_10_ levels reveal important public health concerns. Between 2001 and 2020, the WHO guideline for annual mean PM_10_ concentrations was 20 µg/m^3^ (World Health Organization (WHO) 2005). From 2001 to 2011, virtually all stations with valid data exceeded this threshold, only two annual measurements, both from Leça do Balio – Matosinhos in 2009 and 2011, fell below the guideline. Starting in 2012, a gradual improvement became apparent: the proportion of stations exceeding the 20 µg/m^3^ limit declined over the years, with notable reductions between 2014 and 2020. Still, full compliance remained rare. Under the updated WHO guideline of 15 µg/m^3^ (World Health Organization (WHO) 2021), which reflects the most recent evidence on the health effects of particulate exposure, the situation remains even more critical. In 2021, all six stations with valid data exceeded the 15 µg/m^3^ threshold, and in 2022 none of the 14 stations with valid annual means met the WHO 2021 target. These findings clearly indicate that while regulatory and technological progress has led to reductions in particulate concentrations, ambient PM_10_ levels across the PMA continue to surpass health-based guidelines reinforcing the need for sustained and more stringent air quality management strategies.

The public health implications of these findings are supported by extensive epidemiological literature focused specifically on the PMA. Duarte et al. (2025) reported that the region exhibits higher cardiorespiratory mortality rates compared to other major Portuguese metropolitan areas, with PM_10_ levels significantly associated with this elevated risk. Using spatial analysis and mortality data, they found an average of 202.94 deaths per 100,000 inhabitants in the PMA, surpassing the 169.70 observed in the LMA. Simões et al. (2025) and Brito et al. (2022) further emphasized the disproportionate health burden in northern and coastal Portugal, where long-term exposure to particulate pollution contributes to more than 5000 premature deaths per year. These studies consistently link ambient PM to excess morbidity and mortality, particularly from cardiovascular and respiratory conditions. The convergence of these findings with the present analysis of ambient concentrations strengthens the evidence that the populations of the PMA remain chronically exposed to harmful air pollution, despite overall progress.

Public awareness around air quality is also rising in Portugal, especially in communities directly impacted by industrial emissions or traffic-related pollution. A recent national survey conducted by Canha et al. (2022) highlighted that 61% of residents in an industrialized suburban area rated local air quality as “poor” or “very poor,” compared to only 14% in the general population. This striking perception gap reflects lived experiences of environmental inequality and strengthens public demand for stronger air quality policies. Such perceptions, combined with scientific evidence of health risks, increase pressure on policymakers to implement more effective, transparent and participatory environmental governance.

In light of these findings, it is critical to explore actionable strategies that have proven successful in other metropolitan contexts. For example, significant improvements in air quality have been observed in several Chinese cities following the implementation of broad decarbonization plans, encompassing industrial restructuring, expansion of clean residential energy and reinforced vehicle emissions control (Gao et al. 2023). At a more local scale, mobility-oriented initiatives such as the removal of worn-out vehicles and the development of pedestrian and cycling infrastructure have been recommended to promote active transportation and reduce PM_10_ levels, as demonstrated by Khoshakhlagh et al. (2023). In Portugal, similar approaches are increasingly supported by a strengthened policy and regulatory framework. The updated National Energy and Climate Plan 2030 (PNEC 2030), revised through Parliamentary Resolution No. 127/2025 of 10 April (Portugal 2025a), prioritizes sustainable mobility, modal shift and transport decarbonization, alongside renewable energy expansion and energy efficiency targets (European Alternative Fuels Observatory (EAFO) 2025). These objectives are further reinforced by national incentive programs under the “Mobilidade Verde” scheme, which provide financial support for zero-emission vehicles for both passenger and goods transport, including electric vehicles, vans, cargo bikes and charging infrastructure, accelerating fleet electrification (European Alternative Fuels Observatory (EAFO) 2025). In parallel, recent regulatory instruments, including Decree-Law No. 93/2025 and updated electric mobility regulations issued by the national energy services regulator, establish a comprehensive legal basis for the deployment and governance of electric charging infrastructure (Portugal 2025b; Net Zero Compare 2025). Together, these measures offer transferable and locally relevant pathways for regions like PMA, where the relatively homogeneous pollution patterns observed suggest that broad, region-wide structural interventions may yield substantial air quality benefits.

Urban green infrastructure (GI) has also emerged as a key tool for improving air quality and enhancing population resilience (Herath and Bai 2024). According to Lopes et al. (2025), green spaces in Porto can mitigate urban heat island effects, lower particulate concentrations and provide co-benefits in terms of physical activity, mental health and biodiversity. However, their analysis also revealed unequal distribution of vegetated areas, with socioeconomically disadvantaged neighborhoods often lacking adequate access to green spaces. This spatial mismatch can exacerbate environmental injustice, reinforcing the need for equitable urban planning. GI can also buffer the health impacts of heatwaves and air pollution, particularly when designed to maximize canopy cover, dispersion efficiency and microclimatic benefits. The effectiveness of such interventions, however, depends on strategic integration with transportation planning, building design, and emission control policies.

In summary, while PM_10_ concentrations in the PMA have generally declined over the past two decades, recent years have seen stagnation or increases in some locations. The persistence of exceedances above WHO guideline values, combined with limited spatial differentiation across station types, suggests that exposure remains widespread and warrants continued public health attention. Addressing this challenge will require coordinated efforts involving pollution source mitigation, equitable urban greening and long-term investment in air quality monitoring and public awareness. In this context, ongoing national and European initiatives, such as Portugal’s National Energy and Climate Plan 2030, incentive schemes for clean mobility and the EU’s broader decarbonization strategies, offer a promising pathway to further reduce air pollution levels. With coordinated implementation and continued surveillance, these measures have the potential to deliver meaningful improvements in air quality and public health across the PMA and comparable European urban regions. Nonetheless, this study has some limitations. The analysis was restricted to PM_10_ data, without including other key pollutants such as PM_2.5_ or NO_2_, which often have strong health associations. Additionally, meteorological variables were not explicitly incorporated, despite their known influence on pollutant dispersion and accumulation. Future studies should incorporate meteorological data and other pollutants to enhance understanding of air pollution dynamics and their impacts in the region.

## Conclusions

This study presents a robust two-decade evaluation of PM_10_ concentrations across the PMA, highlighting temporal trends, spatial distribution and differences by monitoring station classification. The results reveal a general decline in annual PM_10_ levels since the early 2000s. However, in recent years, concentrations have plateaued or increased at several sites, and exceedances of WHO guideline values, particularly the updated 2021 threshold of 15 µg/m^3^, remain widespread. No statistically significant differences were found between station classifications or among individual stations within each group, suggesting that PM_10_ exposure is a region-wide issue not limited to isolated pollution hotspots.

The new European Air Quality Directive (EU) 2024/2881 of the European Parliament and of the Council of October 23, 2024 on ambient air quality and cleaner air for Europe, formally adopted in October 2024, sets stricter limit values for inhalable PM_10_, to be met by January 1, 2030. The directive aims to align more closely with the 2021 WHO guidelines, which recommend an annual limit value of 15 µg/m^3^ for PM_10_. The new EU limits are expected to reflect this recommendation, but the exact details will be confirmed in future official publications.

These findings underscore the importance of sustained investment in air quality management, especially in metropolitan regions where population density and diverse emission sources converge. Meeting health-based air quality standards will require the integration of pollution mitigation strategies, urban planning and strengthened environmental surveillance. Continued research is essential to track progress, understand evolving patterns, and support the development of evidence-based public health and environmental policies aimed at reducing population exposure and promoting environmental justice in the PMA and comparable urban contexts.

## Supplementary Information

Below is the link to the electronic supplementary material.

ESM 1 (DOCX 30.1 KB)

## Data Availability

All data used in this study are publicly available from open-access databases. The complete compiled spreadsheet used in the analyses is available from the corresponding author upon reasonable request.

## Abbreviations

COPD: Chronic obstructive pulmonary disease
GI: Urban green infrastructure
LMA: Lisbon Metropolitan Area
PMA: Porto Metropolitan Area
PM_10_: Coarse particulate matter
WHO: World Health Organization

## Declarations

### Clinical trial number

This is not applicable.
