# Supplementary material for: Coarse particulate matter air pollution in Porto Metropolitan area, Portugal: time series analysis, trends and implications for public policies
Source: Environ Sci Pollut Res Int. 2026 Feb 27;33(9):3947–61. doi: 10.1007/s11356-026-37546-w (PMC13043585; doi:10.1007/s11356-026-37546-w)
Supplement: Supplementary file 1 — (DOCX 30.1 KB) [file 11356_2026_37546_MOESM1_ESM.docx]

***Supplemental Material***

**Coarse Particulate Matter Air Pollution in Porto Metropolitan Area, Portugal: Time Series Analysis, Trends and Implications for Public Policies**

| **Table of Contents** | Page |
| --- | --- |
| **Table S1.** Annual mean PM10 concentrations (µg/m^3^) for each of the 15 monitoring stations, based on validated yearly data. Values highlighted in blue are below the WHO air quality guidelines in effect for the respective year. | 2 |
| **Table S2.** | 4 |

**Table S1.** Annual mean PM10 concentrations (µg/m^3^) for each of the 15 monitoring stations, based on validated yearly data. Values highlighted in blue are below the WHO air quality guidelines in effect for the respective year.

| **Number - Air quality stations** |  | **2001** | **2002** | **2003** | **2004** | **2005** | **2006** | **2007** | **2008** | **2009** | **2010** | **2011** |
| --- | --- | --- | --- | --- | --- | --- | --- | --- | --- | --- | --- | --- |
| 6 - Custóias – Matosinhos |  | 50.21 ± 32.76 | 27.60 ± 23.04 | 36.55 ± 23.43 | 40.43 ± 24.22 | 48.16 ± 32.20 | 38.27 ± 22.20 | 35.24 ± 19.72 | 22.87 ± 13.14 | 24.71 ± 16.17 | 27.60 ± 15.22 | 30.20 ± 17.69 |
| 7 - VNTelha – Maia |  | 45.33 ± 28.59 | 31.37 ± 15.75 | 36.88 ± 21.14 | 35.62 ± 19.93 | 38.74 ± 23.18 | 33.25 ± 23.04 | 29.54 ± 16.20 | 25.95 ± 12.93 | 24.52 ± 16.52 | 45.99 ± 22.76 | 45.48 ± 24.09 |
| 11 - Leça do Balio – Matosinhos |  | 54.35 ± 36.95 | 42.88 ± 23.45 | 44.62 ± 27.15 | 34.79 ± 22.83 | 36.02 ± 23.89 | 37.53 ± 28.70 | 38.43 ± 29.27 | 41.58 ± 22.81 | 29.09 ± 17.74 | 26.61 ± 14.82 | 23.86 ± 13.06 |
| 10 - Mindelo – Vila do conde |  | - | - | - | - | - | - | - | - | - | 25.74 ± 14.08 | 29.50 ± 20.97 |
| 13 - Anta – Espinho |  | - | - | - | - | - | - | - | - | - | - | 26.29 ± 16.57 |
| 5 - Emesinde – Valongo |  | 48.95 ± 30.42 | 41.32 ± 21.28 | 41.63 ± 26.67 | 36.58 ± 21.53 | 41.40 ± 24.98 | 39.87 ± 21.95 | 38.49 ± 20.01 | 28.96 ± 14.89 | 30.22 ± 15.65 | 29.01 ± 15.24 | 32.66 ± 19.71 |
| 14 - Sobreiras – Lordelo do Ouro |  | - | - | - | - | - | - | - | - | 24.71 ± 13.25 | 24.94 ± 13.83 | 31.60 ± 18.45 |
| 12 - Burgães – Santo Tirso |  | - | - | - | - | - | - | - | - | 19.50 ± 15.43 | 24.94 ± 13.83 | 11.84 ± 11.29 |
| 8 - Avintes |  | - | - | - | - | - | - | - | - | - | 27.24 ± 16.25 | 25.73 ± 16.14 |
| 9 - D. Manuel II – Vermoim |  | 68.05 ± 41.80 | 42.35 ± 24.64 | 40.87 ± 26.24 | 37.27 ± 24.40 | 41.21 ± 28.16 | 37.68 ± 24.37 | 38.30 ± 25.06 | 25.71 ± 17.57 | 26.28 ± 17.19 | 25.47 ± 16.39 | 31.81 ± 23.63 |
| 4 - Franscico Sá Carneiro – Campanhã |  | 124.08 ± 41.26 | 57.24 ± 27.76 | 46.43 ± 24.57 | 40.74 ± 20.50 | 41.04 ± 21.30 | 36.33 ± 18.50 | 35.14 ± 16.51 | 25.57 ± 12.97 | 27.39 ± 24.30 | 27.92 ± 13.79 | 33.83 ± 18.24 |
| 2 - João Gomes Laranjo – S. Hora |  | 81.53 ± 39.18 | 54.36 ± 28.38 | 47.08 ± 26.23 | 37.22 ± 20.90 | 42.37 ± 23.67 | 40.70 ± 21.79 | 39.59 ± 18.98 | 29.39 ± 16.76 | 33.11 ± 16.47 | 39.93 ± 37.41 | 35.38 ± 18.72 |
| 15 - Moreira Neves – Castelões de Cepeda |  | - | - | - | 43.90 ± 25.16 | 48.40 ± 24.44 | 41.95 ± 21.84 | 36.51 ± 17.06 | 26.07 ± 14.59 | 25.25 ± 10.38 | 25.53 ± 12.47 | 31.59 ± 17.98 |
| 1 - Searas – Matosinhos |  | - | - | - | - | - | - | - | - | - | - | - |
| 3 - Meco – Perafita |  | - | 44.04 ± 17.88 | 44.25 ± 23.14 | 39.22 ± 21.84 | 38.33 ± 22.29 | 35.97 ± 18.95 | 36.82 ± 18.76 | 30.75 ± 17.24 | 31.67 ± 15.84 | 30.21 ± 16.05 | 33.74 ± 18.87 |
|  |  |  |  |  |  |  |  |  |  |  |  |  |
|  |  | **2012** | **2013** | **2014** | **2015** | **2016** | **2017** | **2018** | **2019** | **2020** | **2021** | **2022** |
| 6 - Custóias – Matosinhos |  | 19.39 ± 14.65 | 20.73 ± 13.44 | 14.97 ± 8.56 | 18.34 ± 10.16 | 14.28 ± 5.26 | - | - | - | - | - | 17.16 ± 10.26 |
| 7 - VNTelha – Maia |  | 32.86 ± 18.20 | - | 14.20 ± 13.53 | 16.56 ± 11.22 | 16.19 ± 8.76 | 17.90 ± 9.57 | 17.57 ± 9.30 | 18.81 ± 8.78 | 16.12 ± 7.64 | 15.05 ± 8.11 | 23.55 ± 15.56 |
| 11 - Leça do Balio – Matosinhos |  | 17.55 ± 12.47 | 25.09 ± 16.67 | 16.56 ± 9.02 | 19.64 ± 11.53 | 18.83 ± 11.14 | 14.54 ± 9.65 | 18.99 ± 9.61 | 17.49 ± 9.69 | 18.32 ± 8.48 | 17.92 ± 8.04 | 30.49 ± 17.34 |
| 10 - Mindelo – Vila do conde |  | 23.87 ± 16.54 | 23.81 ± 13.29 | 19.04 ± 12.05 | 17.74 ± 10.87 | 15.50 ± 8.88 | 13.84 ± 8.90 | 13.51 ± 7.47 | 16.75 ± 9.40 | 16.59 ± 7.22 | 16.68 ± 9.00 | 16.82 ± 10.36 |
| 13 - Anta – Espinho |  | 23.22 ± 14.57 | 40.98 ± 20.49 | 30.44 ± 17.93 | 18.50 ± 11.00 | 13.77 ± 10.13 | 11.10 ± 9.03 | 12.00 ± 7.55 | 16.95 ± 9.00 | 19.87 ± 9.02 | - | 22.32 ± 16.02 |
| 5 - Emesinde – Valongo |  | 25.38 ± 14.62 | 24.47 ± 15.25 | 22.45 ± 12.86 | 15.65 ± 10.81 | 22.24 ± 15.80 | 22.40 ± 11.00 | 18.96 ± 9.81 | 24.65 ± 10.30 | - | - | 24.83 ± 13.38 |
| 14 - Sobreiras – Lordelo do Ouro |  | 25.54 ± 16.86 | 22.45 ± 15.16 | 18.22 ± 12.43 | 17.46 ± 12.18 | - | - | - | - | - | - | 22.06 ± 13.59 |
| 12 - Burgães – Santo Tirso |  | 12.77 ± 9.87 | - | 9.97 ± 8.83 | 18.68 ± 11.97 | - | 14.40 ± 8.51 | 18.69 ± 8.31 | 24.00 ± 10.11 | 23.66 ± 7.73 | - | 17.20 ± 10.87 |
| 8 - Avintes |  | 23.49 ± 17.75 | 20.85 ± 12.67 | 12.73 ± 9.46 | 10.66 ± 8.42 | - | 16.59 ± 8.27 | 18.98 ± 10.43 | 18.48 ± 12.12 | 16.33 ± 7.34 | 16.78 ± 0.00 | 21.54 ± 21.33 |
| 9 - D. Manuel II – Vermoim |  | 28.58 ± 22.07 | 20.91 ± 14.55 | 15.42 ± 13.39 | 16.12 ± 11.51 | 12.48 ± 8.41 | 15.49 ± 8.50 | 20.23 ± 9.44 | - | - | - | 18.45 ± 10.81 |
| 4 - Franscico Sá Carneiro – Campanhã |  | 26.59 ± 15.85 | 24.62 ± 12.74 | 18.27 ± 9.10 | 18.98 ± 10.51 | 16.87 ± 7.62 | 19.58 ± 9.68 | 18.64 ± 9.24 | 20.63 ± 6.68 | - | - | 27.07 ± 17.57 |
| 2 - João Gomes Laranjo – S. Hora |  | 26.80 ± 16.70 | 25.19 ± 12.33 | 21.08 ± 9.24 | 20.66 ± 9.14 | 20.42 ± 9.51 | 21.65 ± 10.71 | 18.26 ± 8.91 | 17.10 ± 8.76 | 16.40 ± 9.16 | 15.75 ± 8.25 | 20.06 ± 8.76 |
| 15 - Moreira Neves – Castelões de Cepeda |  | 21.41 ± 13.31 | - | 12.74 ± 6.12 | 20.24 ± 11.57 | 10.54 ± 6.77 | 10.79 ± 7.29 | 9.64 ± 5.30 | 9.71 ± 7.04 | 6.20 ± 5.73 | 21.00 ± 7.87 | - |
| 1 - Searas – Matosinhos |  | - | 21.62 ± 11.66 | - | 19.58 ± 10.92 | 19.90 ± 8.71 | 21.48 ± 10.15 | 21.42 ± 10.08 | 17.18 ± 9.17 | 14.47 ± 11.57 | - | 23.70 ± 14.76 |
| 3 - Meco – Perafita |  | 31.10 ± 17.99 | 28.38 ± 13.83 | 23.70 ± 13.37 | 21.98 ± 11.70 | 17.09 ± 8.28 | 19.14 ± 8.66 | 18.59 ± 7.89 | 21.21 ± 7.07 | 22.05 ± 7.14 | 28.10 ± 16.29 | 57.35 ± 18.30 |

**Table S2.** Percentage of years exceeding WHO annual PM_10_ guideline values and relative trends in PM_10_ concentrations at each air quality monitoring station in the PMA.

| **Number - Air quality stations** | **Classification** | **% of Exceedances WHO guideline values** | **Overall trend** | **Trend from 2001-2011*** | **Trend from 2012-2022** |
| --- | --- | --- | --- | --- | --- |
| 6 - Custóias – Matosinhos | Background - Suburban | 65.00% | -65.82% | -39.85% | -11.49% |
| 7 - VNTelha – Maia | Background - Suburban | 82.35% | -48.05% | 0.35% | -28.34% |
| 11 - Leça do Balio – Matosinhos | Background - Suburban | 63.64% | -43.89% | -56.09% | 73.72% |
| 10 - Mindelo – Vila do conde | Background - Suburban | 46.15% | -34.67% | - | -29.55% |
| 13 - Anta – Espinho | Background - Suburban | 45.45% | -15.10% | - | -3.90% |
| 5 - Ermesinde – Valongo | Background - Urban | 90.00% | -49.28% | -33.29% | -2.15% |
| 14 - Sobreiras – Lordelo do Ouro | Background - Urban | 75.00% | -10.70% | - | -13.61% |
| 12 - Burgães – Santo Tirso | Background - Urban | 36.36% | -11.83% | - | -34.68% |
| 8 - Avintes | Background - Urban | 50.00% | -20.91% | - | -8.31% |
| 9 - D. Manuel II – Vermoim | Traffic- Urban | 78.95% | -72.89% | -53.25% | -35.45% |
| 4 - Franscico Sá Carneiro – Campanhã | Traffic- Urban | 75.00% | -78.19% | -72.74% | 1.79% |
| 2 - João Gomes Laranjo – S. Hora | Traffic- Urban | 86.36% | -75.39% | -56.60% | -25.14% |
| 15 - Moreira Neves – Castelões de Cepeda | Traffic- Urban | 64.71% | -52.17% | -28.04% | -1.95% |
| 1 - Searas – Matosinhos | Industrial- Urban | 50.00% | 9.65% | - | 9.65% |
| 3 - Meco – Parafita | Industrial- Suburban | 85.71% | 30.22% | -23.38% | 84.43% |

*Stations with fewer than four years of valid data within a given sub-period were excluded from the corresponding trend calculations.
